# The way aesthetic needs affects the relationship between aesthetic responsiveness and creativity

**DOI:** 10.1371/journal.pone.0331067

**Published:** 2025-09-02

**Authors:** Xiaoxiao Dou, Yannan Zhang, Xiao Wang, Yan Zhang, Ruifeng Hou

**Affiliations:** College of Arts, Northeastern University, Shenyang, China; Shahid Beheshti University, IRAN, ISLAMIC REPUBLIC OF

## Abstract

Although the study of beauty and its education is expanding, there is still a scarcity of research surrounding the development of our minds and their responses to these emotions. The goal of this study was to look at how the way we respond to beauty connects with creativity, and how our need for beauty influences this connection. A total of 748 university students participated in the study conducted in China. The participants filled out anonymous surveys on a website. The surveys included three tools: the Aesthetic Needs Scale (ANS), the Aesthetic Responsiveness Assessment (AReA), and the Williams Creative Tendency Scale (WCTS). A positive connection was found between all three factors studied. Aesthetic needs play a role in how the assessment of aesthetic responsiveness relates to creativity. This means that people who score high on the aesthetic responsiveness tend to be more creative, especially if they also have strong aesthetic needs.

## Literature review

Past philosophers understood that beauty is closely linked to creativity. Immanuel Kant, in his work “Critique of Judgment,” highlighted how important beauty is for human thinking and creativity. He explained that appreciating beauty-without wanting anything from it-helps us think beyond just practical matters and encourages more advanced creative thinking [1]. According to Kant, the ability to appreciate beauty and the subsequent creative act are manifestations of the same cognitive faculties—those that bridge the sensible and the intellectual. Creativity, thus, emerges not only from logical thought but from an aesthetic capacity to perceive the world in a novel and profound way [2]. According to the study by Welke et al. (2023) [3], people who reacted more intensely to artwork were generally thought to exhibit higher levels of creativity. According to the “aesthetic recycling and creativity” paradigm put forth by Brown et al. (2024) [4], creative outcomes that are meaningful to the group and that resonate are retained when there are significant aesthetic reactions. This interplay between aesthetics and creativity has been widely discussed, yet a comprehensive understanding of how aesthetics influence creativity remains incomplete. This essay aims to explore how aesthetics influences creative thought, arguing that the aesthetic dimension of human experience is both a foundation and a means of releasing creative potential.

### Aesthetic needs

Needs are the basic driving force of human existence and an important trigger for social development, which influences human behaviour and determines and the acquisition of well-being. Starting with basic necessities like food and water, Maslow (1943) outlined a tiered framework for human needs that advances to more sophisticated wants like self-actualization and mental health [5]. Following the satisfaction of fundamental requirements, aesthetic wants arise and are linked to the need for harmony, beauty, and artistic experiences. Maslow proposed that when people’s aesthetic demands are met, they become more emotionally fulfilled and engaged with their environment and activities.

Later, the needs for aesthetics and intellect were added to Maslow’s five-level hierarchy [6]. Beauty needs are about appreciating and seeking things that are attractive, balanced, and well-designed. Fulfilling these needs brings more happiness and harmony to life. From a neuroaesthetic point of view, beauty is closely related to the brain’s reward mechanism, which produces a sense of pleasure in the brain when a person perceives beauty [7]. This includes enjoying art, music, nature, and other forms of creative expression. It’s not just about how things look on the outside, but also about the feelings of comfort and joy that come from experiencing neatness and beauty. Research has demonstrated that humans can detect beauty on a regular basis in their daily lives, and many empirical research have helped people enhance their well-being through beauty [8]. The need for aesthetics has been explored in various contexts, including psychology, art, and environmental studies [9].

Csikszentmihalyi (1997) [10] highlights the importance of flow experiences in creative processes, where individuals become fully immersed in activities that resonate with their aesthetic sensibilities. Engaging with aesthetically pleasing environments can enhance creative thinking and problem-solving abilities, as individuals are inspired by their surroundings. Aesthetic needs are also connected to inner motivation. According to Deci and Ryan’s Self-Determination Theory (2000) [11], meeting inner needs, like aesthetic needs, increases motivation and helps people stay committed to their activities.

While we recognize the significance of aesthetic needs, little effort has been made to assess them. Lundy et al. (2010) [12], pioneered the development of the Desire for Aesthetics Scale (DFAS), which assesses individual aesthetic differences. Similarly, the German version of the DFAS, adapted by Tetzlaff et al. (2024) [13], examines the importance individuals place on beauty across various domains and its connection to personality characteristics such as openness. Recent work to create effective evaluation tools has resulted in the development of the Aesthetic Needs Scale (ANS). Świątek et al. (2024) [14] created the ANS, which looks at how much people care about beauty in three areas: making everyday life beautiful, enjoying art, and appreciating buildings and nature.

Aesthetic needs are an important part of human motivation, shaping behavior, creativity, and emotional health. Satisfying aesthetic needs indirectly contributes to life satisfaction by enhancing emotional regulation [15]. New tools like the Aesthetic Needs Scale and the Desire for Aesthetics Scale have helped researchers better understand the role of aesthetic needs in psychology.

### Aesthetic responsiveness

Responses are ways people react to things around them. Aesthetic responses are about how people feel and think when they see beauty. These responses connect psychology and aesthetics, linking emotions, culture, and personal differences. Responses can be both automatic and intentional, involving physical and mental reactions tied to feelings [16]. In an aesthetic sense, these responses include how people emotionally judge and mentally interpret art or beauty [17]. Csikszentmihalyi’s idea of “flow” (1996) explains that when people deeply focus on beauty-related activities, they feel fully engaged, which improves their aesthetic experiences.

A variety of factors, such as emotions, cultural background, and individual variances, influence aesthetic responses. Positive emotional states have been shown to improve sensitivity to beauty and assist aesthetic experiences (Dewey, 1934) [18]. For instance, people are more likely to react emotionally strongly to beautiful objects when they feel content or at ease. Aesthetic responses are also significantly shaped by cultural context. Hekkert (2006) [19] emphasizes that people’s aesthetic reactions are influenced by the meanings and values of beauty that differ among cultures. For example, whereas some cultures consider art to be the main source of beauty, others may place a higher importance on the beauty found in nature.

A People’s mental health and well-being are strongly correlated with their aesthetic reactions. According to research, people’s sense of wellbeing can be considerably improved by partaking in aesthetic pursuits such enjoying music, art, or outside activities (Kaplan & Kaplan, 1989) [20]. Savoring pleasant experiences can improve mental health, reduce stress, and elevate mood. Additionally, people might use aesthetic reactions as a significant tool for self-expression and identity formation. According to studies, aesthetic encounters have the potential to foster self-expression and self-understanding (Diessner et al. 2008) [21].

Information theory and the dynamic subjective perception of art are further connected in the Van de Cruys et al.(2022) study [22]. They suggest that an aesthetic stimulus’s worth lies not just in its immediate sensory qualities but also in the way it improves our generative models and lowers prediction mistakes. This viewpoint provides a thorough foundation for comprehending aesthetic reaction as an active, productive activity as opposed to a passive admiration of beauty.

### Creativity

One important aspect of human thought is creativity, which is coming up with original and worthwhile concepts or solutions. Although there are many ways to define creativity, it usually involves two primary elements: originality and efficacy. Numerous academics emphasize that creativity encompasses both the ability to come up with fresh ideas and the practicality of those ideas (Runco & Jaeger, 2012) [23]. Psychology research frequently focuses on the traits, processes, and determinants of creativity. Amabile (2018) [24], for instance, put out a social-psychological paradigm that highlights the significance of personal characteristics, the external environment, and intrinsic drive in creative performance.

Creating original and attractive works of art and design is the main task of aesthetic creativity, a subset of creativity. This creative style places a strong emphasis on form, expression, and aesthetics and is frequently impacted by historical, cultural, and individual experiences. Hagman (2005) [25] studied the function of aesthetic creativity in the process of creating art, emphasizing the innate drive and aesthetic sensibility of artists. He pointed out that in addition to technical proficiency, aesthetic creativity also entails a deep comprehension of and capacity for expressing aesthetic experiences.

Emotional experience is regarded as a major contributing factor in the study of artistic creativity. Positive emotional states have been shown to promote inspiration and creativity in artistic work (Oriol et al. 2016) [26]. Furthermore, people’s mental health and self-expression can be improved by the aesthetic experiences that arise during artistic work. The relationship between artistic and creative creativity is a complex subject. With its own distinct traits and requirements, aesthetic creativity can be viewed as a particular expression of creativity. Artists tend to consider beauty from multiple perspectives, which may increase flexibility and divergent thinking, which is at the heart of aesthetic creativity [27].

## Hypotheses

The correlation between aesthetic response and creativity has been noted for a long time, and Nakamura and Csíkszentmihályi’s (2009) ‘Flow Experience Theory’ [28] suggests that when people reach a state of mindfulness while performing an activity, their creativity and efficiency are significantly enhanced. When people appreciate art, natural beauty, or other forms of beauty, the more pronounced the aesthetic response, the easier it is to enter a state of flow, which can help stimulate creative thinking and behaviour. Andreasen’s (2011) study [29] explored the neural basis of creative thinking and psychological perception, and showed that an aesthetic response stimulates an individual’s affective and cognitive resources, which are essential for creative thinking. Through aesthetic experiences and responses, individuals may be more likely to enter a state of openness and acceptance of new ideas, a state conducive to creative thinking. Consequently, this analysis proposes the following hypothesis:

**H1** aesthetic responsiveness is positively related to creativity.

Maslow (1987) [30] discussed in his hierarchy of needs theory that aesthetic needs are a high-level need that involves the realisation of human self-worth. It laid the theoretical foundation for subsequent research on the psychological mechanisms of aesthetic needs. Gerge’s research (2017) [31] demonstrates that aesthetic responses are ‘embodied’, and that profound emotional and perceptual involvement leads to a need for more beautiful stimuli, hence raising aesthetic requirements. Neuroscience has discovered that aesthetic responses differ from survival needs in that high aesthetic responders continually seek artistic beauty, resulting in stable demands [32]. According to Graff and Landwehr’s pleasure-interest model [33], Aesthetic responses and demands are not independent of one another, but rather interact dynamically. Aesthetic responsiveness influences the development of aesthetic needs, and individuals with strong aesthetic responses tend to develop higher aesthetic needs. The broaden-and-build theory by Fredrickson (2001) [34] suggests that the ‘broadening’ effect of pleasant emotions gives a possible explanation for the interplay between aesthetic responses and needs. Williams et al. (2018) [35] conducted a neuroaesthetics study with over 1,000 participants, using resting-state fMRI. Individuals who responded strongly to aesthetics had more functional connectivity in emotion-related brain areas. This led to the creation of stronger aesthetic needs. As a result, we formulated this hypothesis:

**H2** Aesthetic responsiveness is positively related to aesthetic needs.

Deci et al.‘s (2012) Self-Determination Theory [36] emphasizes that individuals achieve higher levels of psychological well-being and creativity when their intrinsic motivations are fulfilled. Aesthetic needs, as a form of intrinsic motivation, drive individuals to invest more emotional and cognitive resources in aesthetic experiences. This active engagement can enhance creative thinking and behavior, thus fostering the development of creativity. Friedman et al. (2001) [37], in his study, explored how motivation affects creativity, and the finding that aesthetic needs can be seen as an intrinsic motivation that drives individuals to seek out experiences of beauty and creative expression supports the positive correlation between aesthetic needs and creativity. That is, when individuals are motivated by a facilitative focus, they are likely to show higher levels of creativity. So, we made this assumption:

**H3** A high aesthetic needs is positively related to creativity.

Furthermore, studies indicate that individuals with high aesthetic sensitivity-those who prioritise aesthetic needs-exhibit greater creativity in problem-solving tasks. For example, Runco and Acar (2012) [38] found that aesthetic responsiveness correlates positively with creative thinking and divergent thinking abilities. This suggests that individuals who actively seek aesthetic experiences are better equipped to generate original ideas and solutions, as their aesthetic needs drive them to explore various perspectives and forms.

The role of aesthetic needs in enhancing creativity is also supported by the concept of intrinsic motivation. Deci’s Self-Determination Theory (2012) [36] posits that satisfying intrinsic needs, including aesthetic needs, leads to greater engagement and persistence in creative endeavours. When individuals are motivated by their aesthetic preferences, they are more likely to experiment and innovate, resulting in heightened creativity.

Moreover, environmental factors significantly influence the relationship between aesthetic needs, aesthetic responses, and creativity. Studies by Hekkert (2014) [39] suggest that exposure to aesthetically rich environments can stimulate creativity by encouraging individuals to engage more deeply with their aesthetic preferences. Strong aesthetic responses inspire creativity, which feeds into the deepening of the aesthetic experience, while the escalation of the aesthetic response fuels the expanding aesthetic need [40]. Therefore, the following expectation was proposed:

**H4** Aesthetic needs plays a mediating role in the relationship between aesthetic responsiveness and creativity.

## Tools

### Aesthetic Needs Scale (ANS)

Dan et al. (2021) [41] developed a scale for assessing students’ aesthetic skills, focusing on four domains: music, painting, literature, and film. Separately, Świątek et al. (2024) [14] created the Aesthetic Needs Scale (ANS), which measures the importance individuals place on beauty across three domains: the aestheticisation of daily life, contact with artistic creation, and the aestheticisation of built and natural environments. The scale was developed using three independent samples to create a 12-item Aesthetic Needs Scale with good construct validity, psychometric features and its relationship with other measures that evaluate how proactive someone is in satisfying their aesthetic needs. A seven-point Likert scale (1 being “strongly disagree,” and 7 being “strongly agree”) was used by respondents to express their ideas. The overall amount of aesthetic requirement is indicated by the sum of all ratings. The greater the respondent’s overall score, the greater their aesthetic requirement. This study used the Aesthetic Needs Scale created by Świątek et al. (2024) [14], which was translated into Chinese using the back-translation method suggested by Brislin (1970) [42] and independently translated by three bilingual specialists. The Chinese version of the scale was then pre-tested on fifty university students to determine its accuracy and clarity. In this study, the dimensions and the overall scale showed high internal consistency reliability. The study found that the aestheticization of daily life (α = 0.85), contact with artistic creations (α = 0.94), aestheticization of the built and natural environments (α = 0.92), and overall internal consistency reliability (α = 0.95) all had high reliability coefficients.

### Aesthetic Responsiveness Assessment (AReA)

Schlotz et al. (2021) [43] designed the original version of this measure to assess people’s aesthetic reaction to art. The initial scale had 18 items in three dimensions, and samples were obtained in the United States and Germany to demonstrate the scale’s utility in various linguistic and cultural contexts. Peng’s (2023) [44] reliability test of the Chinese version of the Aesthetic Responsiveness Scale among college students introduced the Aesthetic Responsiveness Scale to the Chinese college student population for reliability testing, providing a useful tool for aesthetic research in China and filling a gap in the field in China. The revised scale consists of 12 items and 3 dimensions, with the overall structure unchanged and the specific items adjusted. Following the correction, the overall dependability (α = 0.87) was good. more overall scores indicate more aesthetic response. The respondents used a five-point Likert scale to grade the measure, with 1 denoting “strongly disagree” and 5 denoting “strongly agree.” This study used the Chinese version of peng’s (2023) revised Aesthetic Responsiveness Scale. The Cronbach’s alpha coefficient in this study was (α = 0.94) The reliability coefficients of the individual categories and the aggregate scores were high: (α = 0.92) for aesthetic appreciation, (α = 0.91) for aesthetic experience, and (α = 0.82) for aesthetic creation behavior.

### Williams Creative Tendency Scale (WCTS)

The Creative Tendency Scale, designed by Williams (1980) [45], is mainly used to measure an individual’s tendency to be creative. The scale consists of 50 questions, and the scoring method indicates that the total creativity score is proportional to the creative tendency, which includes four dimensions: adventurousness, curiosity, imagination, and challenging. Xu (2021) [46] revised the scale based on the original version, using exploratory factor analysis to filter the questions. Finally, the original four dimensions were retained, with each dimension containing 5–8 questions, for a total of 23 questions. With a reliability range of 0.7 to 0.9, the updated scale’s reliability was acceptable. This study used the Chinese version of the Creative Tendency Scale as revised by Xu (2021) [46]. Using a five-point Likert scale, where “1” represents “strongly disagree” and “5” represents “strongly agree,” respondents scored the updated Xu’s scale. The overall creativity score (α = 0.95), as well as the individual characteristics of adventurousness (α = 0.87), curiosity (α = 0.87), imagination (α = 0.88), and challenge (α = 0.89), all had good reliability coefficients in the current study.

## Study group, procedure and ethics statement

A college advertisement was utilized to recruit participants for this study, which concentrated on young Chinese college students. Each participant received payment for filling out the questionnaire. Prior to beginning the formal survey, an informed consent screen was presented on the online Questionnaire Star platform, and participants were required to confirm their willingness to participate by checking a consent box. All participants were informed of the study’s objectives, procedures, estimated time commitment, the voluntary nature of participation, their right to withdraw at any time, and the confidentiality and anonymity of their responses.

Only adult participants aged 18 and over were included in the study; therefore, no parental or guardian consent was required. The final sample consisted of 748 Chinese university students (male = 566, female = 182), aged between 18 and 35 years (M = 25.42). All participants were right-handed, had normal or corrected-to-normal vision, and reported no history of neurological disorders or color blindness.

This study did not differentiate between individuals with or without formal art training, focusing instead on the general youth population to examine the relationship between aesthetic needs and creativity.

The research process was approved by the Northeastern University Institute of Art’s Research Ethics Committee. Every method was used in compliance with the Declaration of Helsinki. Informed consent was obtained from all participants prior to data collection.

## Results

### Confidence and validity analysis

The validity and reliability of the scale data gathered were confirmed in this study using SPSS 23 and Mplus 28, and measurement models were created using structural equation models [47].

The error variance for the aesthetic responsiveness was between 0.185 and 0.551, with positive and significant results. The standardized regression coefficients ranged from 0.615 to 0.903, and none were close to 1. The standard errors (SEs) of the measurement error variance were between 0.012 and 0.038, with no significant SE found. Similarly, the error variance for aesthetic needs ranged from 0.183 to 0.492, with positive and significant results. The standardized regression coefficients were between 0.713 and 0.904, with none close to 1. The SEs of the measurement error variance were from 0.011 to 0.029, with no major SE seen.

The error variance for creativity was between 0.287 and 0.698, with results that were statistically significant. The standardized regression coefficients ranged from 0.550 to 0.844, with none close to 1. The SEs of the measurement error variances were between 0.017 and 0.033, with no significant SE found.

The fit indices for the three scales were within an acceptable range (as shown in Table 1). Confirmatory factor analysis showed the three scales had good validity [48].

**Table 1:** Fit test data for the scales.

Model	X^2^/df	RMSEA	SRMR	CFI	TLI
Standard	<3	<0.08	<0.05	>0.9	>0.9
AReA	3.314	0.056	0.033	0.974	0.967
ANS	3.885	0.062	0.045	0.928	0.917
WCTS	3.634	0.059	0.05	0.912	0.902

The tests for convergent validity found that the standardized factor loadings for the aesthetic responsiveness, aesthetic needs, and creativity scales were 0.615 to 0.903, 0.713 to 0.904, and 0.550 to 0.844, all above the acceptable threshold of 0.5 and statistically significant [49]. The composite reliability (CR) for each scale dimension was between 0.903 and 0.965, 0.890 and 0.956, and 0.919 and 0.936, all above the threshold of 0.7 [50]. The average variance extracted (AVE) values ranged from 0.481 to 0.705, 0.605 to 0.725, and 0.468 to 0.621, confirming sufficient convergent validity.

The discriminant validity tests showed that the correlation coefficients between dimensions of the three scales were from 0.669 to 0.907, all statistically significant. The square roots of the AVE for each dimension ranged from 0.684 to 0.851, and the correlation coefficients were lower than the square roots of the AVE, showing both good correlation and clear differences between the latent variables [51].

### Descriptive statistics and correlations

The descriptive statistics for each variable are shown in Table 2, together with the correlations, averages, standard deviations, and results of the Shapiro-Wilk test. Significant departures from normalcy were shown by the three variables’ respective W values of 0.95, 0.97, and 0.97, as well as by all related p-values being less than 0.001. According to these findings, the data distributions for every variable show a substantial departure from normalcy at a significance threshold of p < 0.001.

**Table 2:** Mean, standard deviation (SD), Shapiro–Wilk test, and correlations of the study variables.

	M	SD	Shapiro–Wilk test	1	2	3
ANS	87.09	13.44	w = 0.95; p < 0.001***	1		
AReA	41.44	9.56	w = 0.97; p < 0.001***	.612**	1	
WCTS	85.20	15.23	w = 0.97; p < 0.001***	.642**	.698**	1

N = 748. **p < 0.01; ***p < 0.001. ANS = Aesthetic Needs Scale; AReA = Aesthetic Responsiveness Assessment; WCTS = Williams Creative Tendency Scale.

According to the correlation study, there is a substantial Pearson connection between ACN and AReA (0.612), between ACN and WCTS (0.642), and between AReA and WCTS (0.698). The three variables exhibit moderate to strong positive connections, with all correlations being significant (p < 0.01). The strong positive connections shown between ACN, AReA, and WCTS imply that people’s aesthetic experiences may be intimately linked to their aesthetic cognitive need, aesthetic reaction ability, and aesthetic production behavior. These correlations offer a starting point for additional investigation into possible causative or mediated connections between the variables. Future studies can, for example, look at whether aesthetic cognitive requirement drives aesthetic creation behavior directly or whether aesthetic reaction capacity mediates this impact.

### Mediating effect of aesthetic needs

**Fig 1:**
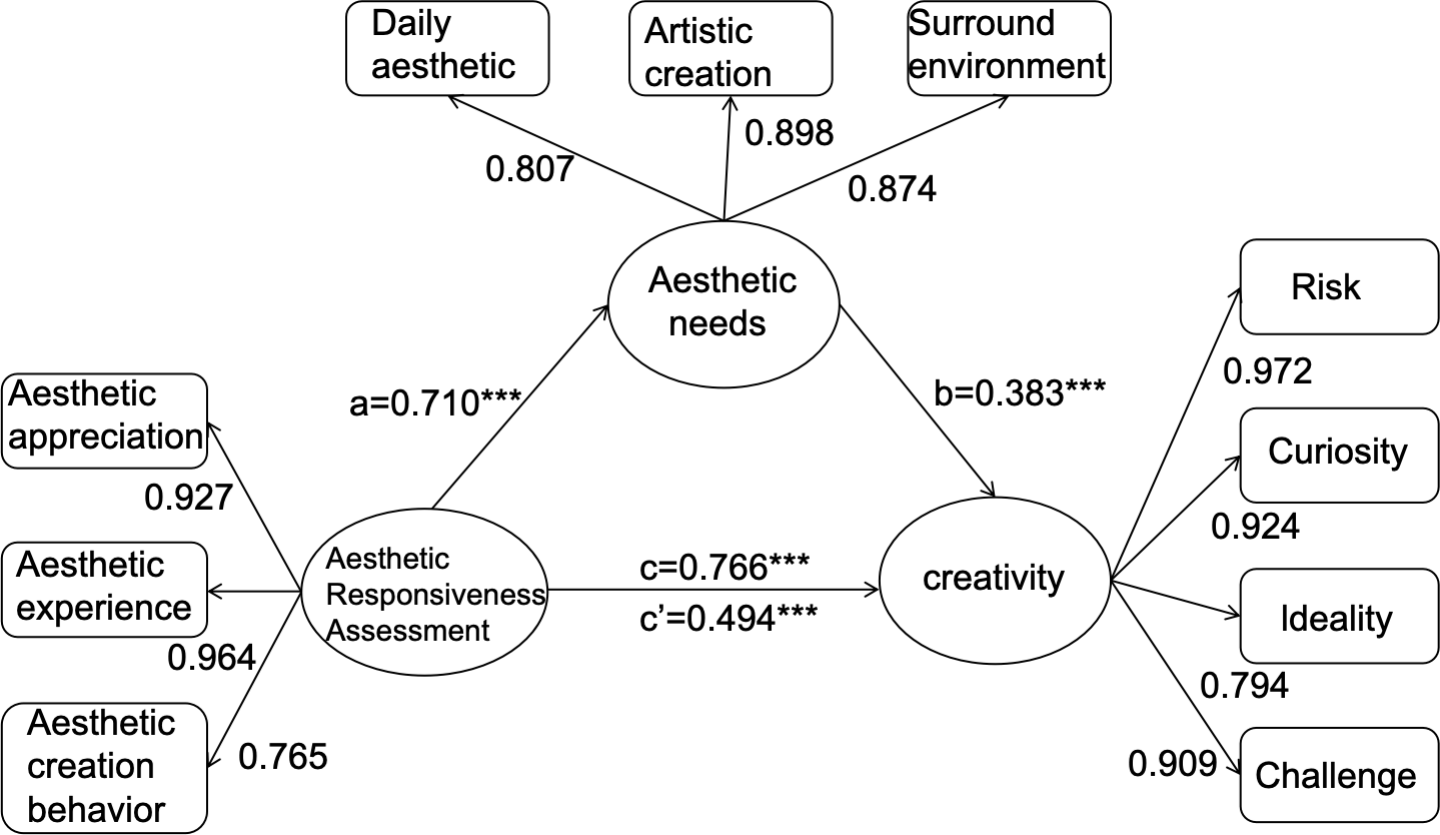
Mediation analysis theoretical model of the mediating role of aesthetic needs in aesthetic responsiveness and creativity. ***p < 0.001.

In order to validate the mediating role of aesthetic needs in aesthetic responsiveness and creativity (H4), a single mediator mediation model Model 4 (Fig 1) was chosen.

According to the investigation, one factor that explained aesthetic needs was the degree of aesthetic responsiveness. This indicates that a high-intensity aesthetic response assessment predicts a higher level of aesthetic needs (path a: β = 0.710; p < 0.001). Furthermore, the degree of creativity was positively predicted by aesthetic needs (path b: β = 0.383; p < 0.001).

In the second phase, based on 748 samples, mediation analysis was conducted, assuming a non-zero 95% CI. The effect of aesthetic responsiveness on creativity was found to be significantly mediated by aesthetic needs, with an indirect effect of 0.272, B (SE) = 0.047 (95% CI: 0.208 ~ 0.370). The total effect of aesthetic responsiveness on creativity (path c: β = 0.766; p < 0.001) decreased to a direct effect of β = 0.494 (p < 0.001) when controlling for aesthetic needs (path c′), indicating partial mediation and supporting Hypothesis 4.

## Discussion

This study found that Chinese aesthetic responsiveness has a significant positive impact on their creativity, affirming H1. The relationship between aesthetic responsiveness and creativity has been widely discussed in the study of positive psychology proposed by Seligman (2014) [52]. Under the stimulation of positive emotions, the influence of aesthetic experience and aesthetic responsiveness on creativity is usually considered positive. Cheung (2019) [53] points out that artistic appreciation can trigger aesthetic reactions, which are also often significantly related to creativity. The results of this study are consistent with the findings of the aforementioned scholars.

This study showed that Chinese aesthetic responsiveness, have a strong positive effect on their aesthetic needs, which supports H2. This aligns with Maslow’s hierarchy of needs, and our findings suggest that individuals with stronger aesthetic responsiveness tend to develop higher aesthetic needs, which subsequently promote deeper engagement in aesthetic experiences. The results are consistent with Graf and Landwehr’s pleasure-interest model [33], aesthetic experiences can elicit cognitive drives—which are intimately linked to higher cognitive processing—as well as their own enjoyable reactions. According to the current study, aesthetic responsiveness promotes creativity by favorably predicting aesthetic needs. In other words, those who are highly susceptible to aesthetics are more likely to have more intense aesthetic needs, which in turn fuel intrinsic aesthetic desire to encourage aesthetic engagement activities and enhance creative performance.

This study confirms H3 and H4, demonstrating that aesthetic needs significantly enhance creativity. Friedman (2001) [37] argued that intrinsic motivations are critical drivers of creative thinking, supporting the notion that individuals with heightened aesthetic needs exhibit greater curiosity and flexibility in problem-solving. This study shows that higher aesthetic needs are linked to more creativity, especially in areas needing emotional expression and visual appeal. The results also have practical uses in schools and workplaces. Programs or spaces that meet aesthetic needs can boost creativity and new ideas. These results agree with Deci and Ryan’s Self-Determination Theory (2000) [11], which says that meeting inner motivations like aesthetic needs helps creativity by increasing focus and effort. People with a strong love for beauty often look for chances to explore, understand, and express it, which helps them think differently and come up with new ideas. For example, showing students art, music, or nature can inspire new thoughts, while well-designed offices can support creative work.

The findings underscore the importance of fostering aesthetic awareness and needs within educational systems. Incorporating aesthetic education programs—such as exposure to art, music, and nature—can promote aesthetic responsiveness and creativity among students. These conclusions, situated within the unique Chinese cultural context, extend the application of the AReA and ANS scales into a new cultural domain.

In addition, although the results of this study show a stable positive relationship between aesthetic responsiveness, aesthetic needs, and creativity, it is important to note that this pattern may not hold true for other groups or contexts. For example, individuals who lack art education or aesthetic experiences, or populations from regions that place a lower value on aesthetics, may exhibit different levels of aesthetic responsiveness or creativity. Additionally, in cultural environments with high levels of pragmatism or instrumental rationality, the effect of aesthetic needs on creativity may be weaker than the results of this study. Future research could further explore these possibilities through comparative or subgroup analyses.

Another concern is that the present study did not distinguish whether subjects had a background in art practice or formal art education. It is unclear whether individuals with regular aesthetic training or in creative industries differ significantly from those without such experience in levels of aesthetic responsiveness, aesthetic needs, and creativity. It is recommended that future research include artistic practice as a potential moderating variable to further test its moderating effect on the pattern of relationships found in this study.

## Limitations

Due to the researcher’s time and capacity constraints, the study’s sample was limited to students selected from a few Chinese universities; additional Chinese college students in other provinces could not be included. This limited the interpretation of the study’s findings and the conclusions that could be made from them.

This study focuses on three factors-aesthetic responsiveness, aesthetic needs, and creativity-that have a significant impact on the aesthetic behavior of Chinese college students. The variables examined in this study do not include other potential contributing factors, such as the personality features of Chinese college students, the economic and cultural standing of their cities, the aesthetic surroundings, the parents’ income level, or their opinions toward art.

This study uses a questionnaire survey to gather data about the personal background variables, aesthetic needs, aesthetic responsiveness, and creativity of Chinese college students. Concerns about whether these subjects can accurately express and reflect the actual situation may skew the study’s findings.

## Supporting information

S1 Table Original data for questionnaire scores, descriptive statistics, and figures presented in the study. (XLSX)
